# Supplementary material for: Exploring physician approaches to conflict resolution in end-of-life decisions in the adult intensive care unit: protocol for a systematic review of qualitative research
Source: BMJ Open. 2022 Jul 21;12(7):e057387. doi: 10.1136/bmjopen-2021-057387 (PMC9310170; doi:10.1136/bmjopen-2021-057387)
Supplement: Supplementary data [file bmjopen-2021-057387supp001.pdf]

**Ovid MEDLINE**

1. exp Terminal Care/
2. Decision Making/
3. Decision Making, Shared/
4. exp Refusal to Treat/
5. Withholding Treatment/
6. Terminally Ill/
7. Palliative Care/
8. end of life.ti,ab,kf.
9. (end adj2 life).ti,ab,kf.
10. (end adj2 life care).ti,ab,kf.
11. EOL*.ti,ab,kf.
12. EOL care.ti,ab,kf.
13. best interest*.ti,ab,kf.
14. withdraw*.ti,ab,kf.
15. withhold*.ti,ab,kf.
16. decision*.ti,ab,kf.
17. resol*.ti,ab,kf.
18. consent.ti,ab,kf.
19. refusal*.ti,ab,kf.
20. Intensive Care Units/
21. critical care*.ti,ab,kf.
22. intensive care*.ti,ab,kf.
23. adult ICU.ti,ab,kf.
24. AICU.ti,ab,kf.
25. ICU*.ti,ab,kf.
26. "Dissent and Disputes"/
27. Negotiating/
28. Consensus/
29. conflict*.ti,ab,kf.
30. dispute*.ti,ab,kf.
31. disagreement*.ti,ab,kf.
32. dissent*.ti,ab,kf.
33. Physicians/
34. physician*.ti,ab,kf.
35. doctor*.ti,ab,kf.
36. nurse*.ti,ab,kf.
37. healthcare professional*.ti,ab,kf.
38. (health adj2 care professional).ti,ab,kf.
39. clinician*.ti,ab,kf.
40. famil*.ti,ab,kf.
41. relative*.ti,ab,kf.
42. therap*.ti,ab,kf.
43. surrogate*.ti,ab,kf.
44. prox*.ti,ab,kf.
45. patient*.ti,ab,kf.
46. Qualitative Research/
47. qualitative.ti,ab,kf.
48. interview*.ti,ab,kf.
49. focus group*.ti,ab,kf.
50. survey*.ti,ab,kf.
51. questionnaire*.ti,ab,kf.
52. empirical*.ti,ab,kf.
53. framework*.ti,ab,kf.
54. 1 or 2 or 3 or 4 or 5 or 6 or 7 or 8 or 9 or 10 or 11 or 12 or 13 or 14 or 15 or 16 or 17 or 18 or 19
55. 20 or 21 or 22 or 23 or 24 or 25
56. 26 or 27 or 28 or 29 or 30 or 31 or 32
57. 33 or 34 or 35 or 36 or 37 or 38 or 39 or 40 or 41 or 42 or 43 or 44 or 45
58. 46 or 47 or 48 or 49 or 50 or 51 or 52 or 53
59. 54 and 55 and 56 and 57 and 58

**Ovid EMBASE**

1. exp Terminal Care/
2. Decision Making/
3. Decision Making, Shared/
4. exp Patient Abandonment/
5. Treatment Withdrawal/
6. Terminally Ill Patient/
7. Palliative Therapy/
8. end of life.ti,ab,kf.
9. (end adj2 life).ti,ab,kf.
10. (end adj2 life care).ti,ab,kf.
11. EOL*.ti,ab,kf.
12. EOL care.ti,ab,kf.
13. best interest*.ti,ab,kf.
14. withdraw*.ti,ab,kf.
15. withhold*.ti,ab,kf.
16. decision*.ti,ab,kf.
17. resol*.ti,ab,kf.
18. consent.ti,ab,kf.
19. refusal*.ti,ab,kf.
20. Intensive Care Unit/
21. critical care*.ti,ab,kf.
22. intensive care*.ti,ab,kf.
23. adult ICU.ti,ab,kf.
24. AICU.ti,ab,kf.
25. ICU*.ti,ab,kf.
26. Conflict/
27. Negotiation/
28. Consensus/
29. conflict*.ti,ab,kf.
30. dispute*.ti,ab,kf.
31. disagreement*.ti,ab,kf.
32. dissent*.ti,ab,kf.
33. Physician/
34. physician*.ti,ab,kf.
35. doctor*.ti,ab,kf.
36. nurse*.ti,ab,kf.
37. healthcare professional*.ti,ab,kf.
38. (health adj2 care professional).ti,ab,kf.
39. clinician*.ti,ab,kf.
40. famil*.ti,ab,kf.
41. relative*.ti,ab,kf.
42. therap*.ti,ab,kf.
43. surrogate*.ti,ab,kf.
44. prox*.ti,ab,kf.
45. patient*.ti,ab,kf.
46. Qualitative Research/
47. qualitative.ti,ab,kf.
48. interview*.ti,ab,kf.
49. focus group*.ti,ab,kf.
50. survey*.ti,ab,kf.
51. questionnaire*.ti,ab,kf.
52. empirical*.ti,ab,kf.
53. framework*.ti,ab,kf.
54. 1 or 2 or 3 or 4 or 5 or 6 or 7 or 8 or 9 or 10 or 11 or 12 or 13 or 14 or 15 or 16 or 17 or 18 or 19
55. 20 or 21 or 22 or 23 or 24 or 25
56. 26 or 27 or 28 or 29 or 30 or 31 or 32
57. 33 or 34 or 35 or 36 or 37 or 38 or 39 or 40 or 41 or 42 or 43 or 44 or 45
58. 46 or 47 or 48 or 49 or 50 or 51 or 52 or 53
59. 54 and 55 and 56 and 57 and 58

**Ovid PsycINFO**

1. Decision Making/
2. exp Treatment Refusal/
3. Treatment Withholding/
4. Treatment Termination/
5. Terminally Ill Patients/
6. Palliative Care/
7. end of life.tw.
8. (end adj2 life).tw.
9. (end adj2 life care).tw.
10. EOL*.tw.
11. EOL care.tw.
12. best interest*.tw.
13. withdraw*.tw.
14. withhold*.tw.
15. decision*.tw.
16. resol*.tw.
17. consent.tw.
18. refusal*.tw.
19. Intensive Care/
20. critical care*.tw.
21. intensive care*.tw.
22. adult ICU.tw.
23. AICU.tw.
24. ICU*.tw.
25. Conflict/
26. Conflict Resolution/
27. Negotiation/
28. conflict*.tw.
29. dispute*.tw.
30. disagreement*.tw.
31. dissent*.tw.
32. Physicians/
33. physician*.tw.
34. doctor*.tw.
35. nurse*.tw.
36. healthcare professional*.tw.
37. (health adj2 care professional).tw.
38. clinician*.tw.
39. famil*.tw.
40. relative*.tw.
41. therap*.tw.
42. surrogate*.tw.
43. prox*.tw.
44. patient*.tw.
45. Qualitative Methods/
46. qualitative.tw.
47. interview*.tw.
48. focus group*.tw.
49. survey*.tw.
50. questionnaire*.tw.
51. empirical*.tw.
52. framework*.tw.
53. 1 or 2 or 3 or 4 or 5 or 6 or 7 or 8 or 9 or 10 or 11 or 12 or 13 or 14 or 15 or 16 or 17 or 18
54. 19 or 20 or 21 or 22 or 23 or 24
55. 25 or 26 or 27 or 28 or 29 or 30 or 31
56. 32 or 33 or 34 or 35 or 36 or 37 or 38 or 39 or 40 or 41 or 42 or 43 or 44
57. 45 or 46 or 47 or 48 or 49 or 50 or 51 or 52
58. 53 and 54 and 55 and 56 and 57

**Web of Science**

((((TS=(terminal care OR decision$making OR end NEAR/2 life OR end NEAR/2 life care OR palliative care OR terminal* ill* OR best interest* OR withdraw* OR withhold* OR decision* OR resol* OR consent OR refusal* OR EOL* OR EOL care))

AND TS=(dissent* OR dispute* OR conflict* OR disagreement* OR negotiat* OR consensus))

AND TS=(physician* OR doctor* OR nurse* OR health$care professional* OR clinician* OR famil* OR relative* OR therap* OR surrogate* OR prox* OR patient*))

AND TS=(intensive care* OR critical care* OR adult ICU* OR ICU* OR AICU))

AND TS=(qualitative* OR interview* OR focus group* OR survey* OR questionnaire* OR empirical* OR framework*)

**Scopus**

(TITLE-ABS-KEY(terminal care) OR TITLE-ABS-KEY(decision making) OR TITLE-ABS-KEY(end W/2 life) OR TITLE-ABS-KEY(end W/2 life care) OR TITLE-ABS-KEY(palliative care) OR TITLE-ABS-KEY(terminal* ill*) OR TITLE-ABS-KEY(best interest*) OR TITLE-ABS-KEY(withdraw*) OR TITLE-ABS-KEY(withhold*) OR TITLE-ABS-KEY(decision*) OR TITLE-ABS-KEY(resol*) OR TITLE-ABS-KEY(consent) OR TITLE-ABS-KEY(refusal*) OR TITLE-ABS-KEY(EOL*) OR TITLE-ABS-KEY(EOL care))

AND (TITLE-ABS-KEY(intensive care*) OR TITLE-ABS-KEY(critical care*) OR TITLE-ABS-KEY(adult ICU*) OR TITLE-ABS-KEY(ICU*) OR TITLE-ABS-KEY(AICU))

AND (TITLE-ABS-KEY(dissent*) OR TITLE-ABS-KEY(dispute*) OR TITLE-ABS-KEY(conflict*) OR TITLE-ABS-KEY(disagreement*) OR TITLE-ABS-KEY(consensus*) OR TITLE-ABS-KEY(negotiat*))

AND (TITLE-ABS-KEY(physician*) OR TITLE-ABS-KEY(doctor*) OR TITLE-ABS-KEY(nurse*) OR TITLE-ABS-KEY(healthcare professional*) OR TITLE-ABS-KEY(health care professional*) OR TITLE-ABS-KEY(clinician*) OR TITLE-ABS-KEY(famil*) OR TITLE-ABS-KEY(relative*) OR TITLE-ABS-KEY(therap*) OR TITLE-ABS-KEY(surrogate*) OR TITLE-ABS-KEY(prox*) OR TITLE-ABS-KEY(patient*))

AND (TITLE-ABS-KEY(qualitative*) OR TITLE-ABS-KEY(interview*) OR TITLE-ABS-KEY(focus group*) OR TITLE-ABS-KEY(survey*) OR TITLE-ABS-KEY(questionnaire*) OR TITLE-ABS-KEY(empirical*) OR TITLE-ABS-KEY(framework*))

**CINAHLS**

(MH “Terminal Care+” OR MH “Decision Making+” OR MH “Refusal to Treat+” OR MH “Treatment Refusal+” OR MH “Euthanasia, Passive” OR MH “Terminally Il Patients” OR MH “Palliative Care” OR TI (“end of life” OR “end W2 life” OR “end W2 life care” OR “EOL*” OR “EOL care” OR “best interest*” OR “withdraw*” OR “withhold*” OR “decision*” OR “resol*” OR “consent” OR “refusal*”) OR AB (“end of life” OR “end W2 life” OR “end W2 life care” OR “EOL*” OR “EOL care” OR “best interest*” OR “withdraw*” OR “withhold*” OR “decision*” OR “resol*” OR “consent” OR “refusal*”))

AND (MH “Critical Care” OR TI (“critical care*” OR “intensive care*” OR “adult ICU” OR “AICU” OR “ICU*”) OR AB (“critical care*” OR “intensive care*” OR “adult ICU” OR “AICU” OR “ICU*”))

AND (MH "Conflict Management“ OR MH “Negotiation” OR MH “Consensus” OR TI (“conflict*” OR “dispute*” OR “disagreement*” OR “dissent*”) OR AB (“conflict*” OR “dispute*” OR “disagreement*” OR “dissent*”))

AND (MH “Physicians+” OR TI (“physician*” OR “doctor*” OR “nurse*” OR “healthcare professional*” OR “health W2 care professional” OR “clinician*” OR “famil*” OR “relative*” OR “therap*” OR “surrogate*” OR “prox*” OR “patient*”) OR AB (“physician*” OR “doctor*” OR “nurse*” OR “healthcare professional*” OR “health W2 care professional” OR “clinician*” OR “famil*” OR “relative*” OR “therap*” OR “surrogate*” OR “prox*” OR “patient*”))

AND (MH “Qualitative Studies” OR TI (“qualitative” OR “interview*” OR “focus group*” OR “survey*” OR “questionnaire*” OR “empirical*” OR “framework*”) OR AB (“qualitative” OR “interview*” OR “focus group*” OR “survey*” OR “questionnaire*” OR “empirical*” OR “framework*”))

**Project MUSE**

(terminal* OR decision* OR making OR end OR life OR care OR palliative OR ill* OR best interest* OR withdraw* OR withhold* OR resol* OR consent OR refusal* OR EOL*)

AND (dissent* OR dispute* OR conflict* OR disagreement* OR negotiat* OR consensus)

AND (physician* OR doctor* OR nurse* OR health* OR professional* OR clinician* OR famil* OR relative* OR therap* OR surrogate* OR prox* OR patient*)

AND (intensive OR critical OR care OR unit* OR adult* OR ICU* OR AICU)

AND (qualitative* OR interview* OR "focus group*" OR survey* OR questionnaire* OR empirical* OR framework*)

**LILACS**

("Terminal Care/" OR "Decision Making/" OR "Refusal to Treat/" OR "Withholding Treatment/" OR "Terminally Ill/" OR "Palliative Care/" OR terminal$ OR decision$ OR making OR end OR life OR care OR palliative OR ill$ OR best interest$ OR withdraw$ OR withhold$ OR resol$ OR consent OR refusal$ OR EOL$) AND ("Conflict Resolution/" OR "Conflict/" OR dissent$ OR dispute$ OR conflict$ OR disagreement$ OR negotiat$ OR consensus) AND ("Physician/" OR physician$ OR doctor$ OR nurse$ OR health$ OR professional$ OR clinician$ OR famil$ OR relative$ OR therap$ OR surrogate$ OR prox$ OR patient$) AND ("Intensive Care/" OR "Intensive Care Unit/" OR intensive OR critical OR care OR unit$ OR adult$ OR ICU$ OR AICU) AND ("Qualitative/" OR qualitative$ OR interview$ OR focus OR group$ OR survey$ OR questionnaire$ OR empirical$ OR framework$) [Subject descriptor]

OR ("Terminal Care/" OR "Decision Making/" OR "Refusal to Treat/" OR "Withholding Treatment/" OR "Terminally Ill/" OR "Palliative Care/" OR terminal$ OR decision$ OR making OR end OR life OR care OR palliative OR ill$ OR best interest$ OR withdraw$ OR withhold$ OR resol$ OR consent OR refusal$ OR EOL$) AND ("Conflict Resolution/" OR "Conflict/" OR dissent$ OR dispute$ OR conflict$ OR disagreement$ OR negotiat$ OR consensus) AND ("Physician/" OR physician$ OR doctor$ OR nurse$ OR health$ OR professional$ OR clinician$ OR famil$ OR relative$ OR therap$ OR surrogate$ OR prox$ OR patient$) AND ("Intensive Care/" OR "Intensive Care Unit/" OR intensive OR critical OR care OR unit$ OR adult$ OR ICU$ OR AICU) AND ("Qualitative/" OR qualitative$ OR interview$ OR focus OR group$ OR survey$ OR questionnaire$ OR empirical$ OR framework$) [Title words]
