# Supplementary material for: Exploring physician approaches to conflict resolution in end-of-life decisions in the adult intensive care unit: protocol for a systematic review of qualitative research
Source: BMJ Open. 2022 Jul 21;12(7):e057387. doi: 10.1136/bmjopen-2021-057387 (PMC9310170; doi:10.1136/bmjopen-2021-057387)
Supplement: Author's manuscript [file bmjopen-2021-057387.draft_revisions.pdf]

BMJ Open is committed to open peer review. As part of this commitment we make the peer review history of every article we publish publicly available.

When an article is published we post the peer reviewers' comments and the authors' responses online. We also post the versions of the paper that were used during peer review. These are the versions that the peer review comments apply to.

The versions of the paper that follow are the versions that were submitted during the peer review process. They are not the versions of record or the final published versions. They should not be cited or distributed as the published version of this manuscript.

BMJ Open is an open access journal and the full, final, typeset and author-corrected version of record of the manuscript is available on our site with no access controls, subscription charges or pay-per-view fees (http://bmjopen.bmj.com).

If you have any questions on BMJ Open's open peer review process please email info.bmjopen@bmj.com

BMJ Open

Protocol for a Systematic Review of Qualitative Research Exploring Physician Approaches to Conflict Resolution in End-of-Life Decisions in the Adult Intensive Care Unit

Journal:	*BMJ Open*
Manuscript ID	bmjopen-2021-057387
Article Type:	Protocol
Date Submitted by the Author:	16-Sep-2021
Complete List of Authors:	Johal, Harleen; University of Bristol Centre for Ethics in Medicine, Bristol Medical School<br>Birchley, Giles; University of Bristol Centre for Ethics in Medicine<br>Huxtable, Richard; University of Bristol Centre for Ethics in Medicine
Keywords:	INTENSIVE & CRITICAL CARE, MEDICAL ETHICS, Adult intensive & critical care < INTENSIVE & CRITICAL CARE, Adult palliative care < PALLIATIVE CARE, PALLIATIVE CARE, Adult intensive & critical care < ANAESTHETICS

SCHOLARONE™
Manuscripts

Harleen Kaur Johal, Giles Birchley & Richard Huxtable

Protocol for a Systematic Review of Qualitative Research Exploring Physician Approaches to Conflict Resolution in End-of-Life Decisions in the Adult Intensive Care Unit

Authors

- Harleen Kaur Johal (***Corresponding Author***)
  Centre for Ethics in Medicine, Population Health Sciences, Bristol Medical School, University of Bristol
  (E-mail: harleen.johal@bristol.ac.uk)
- Giles Birchley
  Centre for Ethics in Medicine, Population Health Sciences, Bristol Medical School, University of Bristol
  (E-mail: giles.birchley@bristol.ac.uk)
- Richard Huxtable
  Centre for Ethics in Medicine, Population Health Sciences, Bristol Medical School, University of Bristol
  (E-mail: r.huxtable@bristol.ac.uk)

Registration

PROSPERO CRD42021193769

Word Count

3643 words

For peer review only

For peer review only - http://bmjopen.bmj.com/site/about/guidelines.xhtml

Harleen Kaur Johal, Giles Birchley & Richard Huxtable

ABSTRACT

Introduction

Conflict is unfortunately well-documented in the adult intensive care unit (AICU). In the context of end-of-life (EOL) decision-making (i.e. the withdrawal or withholding of life-sustaining treatment), conflict commonly occurs when a consensus cannot be reached between the healthcare team and the patient's family on the "best interests" of the critically ill, incapacitated patient. Whilst existing literature has identified potential methods for conflict resolution, it is less clear how these approaches are perceived and utilised by stakeholders in the EOL decision-making process. We aim to explore this by systematically reviewing and synthesising the published evidence, which addresses the following research question: what does existing qualitative research reveal about physician approaches to addressing conflict arising in EOL decisions in the AICU?

Methods and Analysis

Peer-reviewed qualitative studies (retrieved from MEDLINE, Project Muse, EMBASE, Web of Science, PsycINFO, CINAHL and LILACS) examining conflict and dispute resolution in the context of EOL decisions in the AICU setting will be included. Two reviewers will independently screen either all or a randomly selected sample of studies, with a third reviewer independently screening studies of uncertain eligibility. The 'thematic synthesis' approach will be employed to analyse the resulting data (Thomas & Harden, 2008). The quality of included papers will be assessed using the 2018 Mixed-Methods Assessment Tool (MMAT). The GRADE-CERQual approach will be utilised to assess our confidence in the findings.

Ethics and Dissemination

Ethical approval is not required for this review, as only published data will be included. We anticipate that the findings will be of interest to healthcare professionals working in intensive care units and individuals working in bioethics, given the ethically contentious nature of EOL decisions. The findings will be disseminated at both academic conferences and through open-access publication in a peer-reviewed journal. The protocol has been registered on the PROSPERO database (CRD42021193769).

300 words

For peer review only

For peer review only - http://bmjopen.bmj.com/site/about/guidelines.xhtml

ARTICLE SUMMARY

Strengths and Limitations of This Study

- This systematic review is the first to synthesise qualitative research exploring physician approaches to resolving end-of-life conflicts within the adult intensive care unit.
- Given both the bioethical and clinical nature of the research question, this search strategy benefits from utilising both philosophical and clinical bibliographic databases.
- The exclusion of other healthcare professionals' approaches (such as nurses) to resolving conflict may result in effective methods not being identified.
- However, by focusing on qualitative data, rather than theoretical or normative research, we will be able to synthesise methods that physicians actually use to try and resolve conflicts. The findings of this review will therefore better represent the reality and feasibility of clinical practice.

For peer review only

For peer review only - http://bmjopen.bmj.com/site/about/guidelines.xhtml

INTRODUCTION

Conflict is unfortunately well-documented in the adult intensive care unit (AICU). In the context of end-of-life (EOL) decision-making (i.e. the withdrawal or withholding of life-sustaining treatment), it commonly occurs when a consensus cannot be reached on the best interests of the critically ill, incapacitated patient. (1) Families, as surrogate decision-makers, may favour preservation of life, whilst physicians may be reluctant to provide life-sustaining treatment, which is potentially inappropriate. (2) The decision-making process for healthcare professionals (HCPs) is informed by their understanding of the law and professional guidance. In England and Wales, when there is an agreement on the best interests of a patient, there is no need to make an application to the court to withdraw or withhold life-sustaining treatment. (3) Involvement of the courts is however necessitated where disagreement between the conflicting parties has become intractable. This adversarial process exacerbates distress amongst HCPs and families, potentially leading to breakdowns in therapeutic relationships. (4) This arguably justifies the utilisation of alternative methods of conflict resolution, before a dispute becomes sufficiently entrenched to require legal intervention. (1, 5)

There is an extensive body of literature recognising the incidence of conflict in adult ICUs. The 'Conflicus' study surveyed the experiences of 7498 ICU staff members in 323 ICUs in 24 countries. Nurse-physician conflicts were most common (32.6%) and staff-relative conflicts accounted for 26.2% of perceived conflicts. Lack of psychological support and problems with the decision-making process were identified as causes of conflict in EOL care. (6) Indeed, in a critical literature review exploring doctors' and nurses' EOL decision-making process, Flannery and colleagues suggested that more comprehensive and standardised approaches are needed to support all HCPs in making these difficult decisions. (7) One could extend this suggestion into a need for more guidance, to support HCPs in the resolution of disputes when they occur. Whilst academic literature has identified potential routes for dispute resolution, for example, clinical ethics committees and mediation, (8) it is less clear how these approaches are perceived and utilised by stakeholders in the EOL decision-making process, such as healthcare professionals, patients and surrogate decision-makers. (9) It has also been observed in other areas of healthcare that theoretical discussion of ethical challenges is not necessarily representative of the ethical issues that arise in real clinical practice. (10) Further consideration should therefore be given to how these stakeholders actually approach conflict and how they perceive strategies for conflict resolution.

We seek to explore this by systematically reviewing and synthesising the published qualitative evidence on physician approaches to conflict resolution in EOL decisions in the AICU. A coherent and cohesive understanding of current approaches to conflict resolution will provide a starting point for an evidence-base, from which healthcare professionals can be trained in how to approach conflict. (11) The rationale for this review is further supported by recent developments in the field of bioethics, where the value of

For peer review only - http://bmjopen.bmj.com/site/about/guidelines.xhtml

empirical evidence in evaluating normative claims has been increasingly recognised. (12) Hence, in order to address the question of how conflict should be resolved, we must first consider the context of conflict and how physicians attempt to resolve it, so the resulting ethical analysis is informed by and more relevant to clinical practice. (13)

This systematic review shall lay the foundations of a larger empirical bioethics study, in which disagreements between healthcare professionals and patient representatives will be explored. Ultimately, the development of guidelines to support all healthcare professionals, patients and families when conflict arises, will aim to improve their experience during an ethically contentious and emotionally strenuous time. (14)

For peer review only

Aims

This systematic review protocol has been guided by the Preferred Reporting Items for Systematic Reviews and Meta-Analyses Protocols (PRISMA-P) checklist (see online supplementary file), (15) and the review will aim to address the following research question: what does existing qualitative research reveal about physician approaches to addressing conflict arising in EOL decisions (specifically, the withdrawal and withholding of life-sustaining treatments) in the AICU? The standard "Participants, Interventions, Comparators and Outcomes" (PICO) system used in reviews of clinical studies has been modified here, as it is less suited to qualitative evidence synthesis, by using Strech and colleagues' "Methodology, Issue, Participants" (MIP) system. (16)

METHODS AND ANALYSIS

Eligibility criteria

The purpose of this review is to identify studies in which physicians have described their strategies for approaching and resolving conflict around EOL decisions in the AICU. The inclusion and exclusion criteria, following Strech et al's MIP system, are shown in table 1. Peer-reviewed qualitative studies examining conflict resolution in the context of EOL decisions in the adult intensive care setting will therefore be included.

Studies identifying factors, which contribute to the development of conflict in AICU around EOL decisions, will not be included. Identification of these factors is important in the development of conflict resolution strategies, however this would constitute a separate systematic review. Studies relating to conflict around EOL decisions for critically ill children and neonates will also be excluded. Whilst there may be similar circumstances to those which present in an adult intensive care environment, where the patient is incapacitated and a decision must therefore be made in their best interests, there are legal and

For peer review only - http://bmjopen.bmj.com/site/about/guidelines.xhtml

clinical differences between adults and children. The role of the "family and others" is notably different in best interests decision-making processes for adults,(17) as compared to children; and the resulting conflicts may be different in nature. Whilst conflict resolution strategies in AICU could be applied in PICU and vice versa, it is beyond the scope of this review to consider two clinical contexts, with potentially crucial differences, and two groups of stakeholders with differing roles.

Table 1: Inclusion and Exclusion Criteria

	**Inclusion Criteria**	**Exclusion Criteria**
**Context**	Critical/intensive care settings where EOL decisions are made for adults will be included.<br>The terms 'critical' and 'intensive' are often used interchangeably for clinical settings in which patients are seriously ill and/or require some form of life-sustaining treatment.	Clinical settings, in which EOL decision are made for children or neonates will be excluded.<br>Non-critical/intensive care settings will be excluded.<br>Other specialities or settings, in which EOL decisions are made (e.g. community palliative care), will be excluded.
**Methodologies**	Qualitative studies examining conflict resolution in the AICU (e.g. interviews and focus groups).<br>Mixed-methods studies (e.g. surveys with open questions) will also be included for consideration of the qualitative evidence presented.	Non-empirical studies examining conflict resolution. This may include normative and theoretical literature. These have been excluded as we are interested in understanding the lived reality of conflict resolution in clinical practice.<br>Quantitative studies examining conflict resolution in the AICU. These have been excluded as we are interested in deeper exploration of experiences in the AICU, which is not possible using quantitative methods.
**Issues**	Qualitative studies exploring conflict resolution strategies within	Literature that does not explore conflict, dispute, disagreement,

For peer review only

	the AICU, around EOL decisions, will be included.<br>EOL decisions are here defined as decisions relating to the withdrawal or withholding of life-sustaining treatment (e.g. dialysis, clinically-assisted nutrition and hydration, artificial ventilation) or decisions relating to cardiopulmonary resuscitation.<br>Conflict is here defined as a failure to reach an agreement on whether life-sustaining treatment should be withdrawn or withheld. The terms 'conflict', 'dispute', 'disagreement', 'dissent' and 'refusal' are often used interchangeably in the existing literature, and we are therefore interested in studies which explore any of these issues.	dissent, and refusal will be excluded.<br>Literature that does not explore withdrawal or withholding of life-sustaining treatment will be excluded.<br>Empirical studies examining factors, which contribute to conflict, will be excluded. Whilst the identification of factors which cause conflict is fundamental to the development of conflict resolution strategies, it is beyond the scope of this systematic review.
**Participants**	Studies which explore physician approaches to conflict resolution will be included.<br>Studies which explore other stakeholders' perceptions of physician approaches to conflict resolution will also be included.<br>Stakeholders are broadly-defined in three categories: (i) healthcare professionals (e.g. physicians/doctors, nurses and therapists), (ii) adult patients and (iii) patient representatives (e.g. families, spouses, relatives and other surrogate decision-makers).	Literature that does not encompass discussion of stakeholders' (as defined in the inclusion criteria) approaches to conflict in EOL decisions in the AICU, will be excluded.<br>Literature that discusses non-physician led approaches to conflict resolution, e.g. clinical ethics consultation or independent mediation, will be excluded.

For peer review only - http://bmjopen.bmj.com/site/about/guidelines.xhtml

**Timeframe**	Any studies published after 1980 will be included; this is dependent on the commencement dates of databases included in the search.	Studies published before 1980 will be excluded, as they are less likely to be relevant to current clinical practice.
**Types of Publications**	Peer-reviewed journal publications of empirical research.<br>Publications in English.<br>International publications will be included.	Unpublished and grey literature, theses and dissertations, and any published sourced that do not contain empirical studies will be excluded.<br>Publications not in English will be excluded.<br>Conference abstracts will be excluded.<br>Review articles relating to the research question will be used for the identification of empirical studies only.<br>Study authors will be contacted if we are unable to obtain the full text through the university subscription. If the study authors do not respond to this request within two weeks, the study will be excluded.

Search strategy

Scoping searches were conducted, using MEDLINE Subject Headings (MeSH) and truncations of keywords obtained from previous systematic reviews of EOL care in the AICU. (18) The search terms were then combined, using the Boolean operators 'AND' and 'OR'. (19) The MEDLINE search strategy (seen online supplementary file), will be amended for other databases as needed.

Information sources

We will use the following databases to retrieve studies: MEDLINE, Project Muse, EMBASE, Web of Science, PsycINFO, CINAHL and LILACS. These databases have been chosen as they are known to be directly relevant to studies conducted in medicine, social sciences and bioethics. We have imposed

For peer review only - http://bmjopen.bmj.com/site/about/guidelines.xhtml

a restriction on publication dates, as critical care is a rapidly developing field and studies pre-1980 are likely to be outdated. We will also only include papers in English. Due to the resource-intensive nature of reviewing grey literature, it will also be excluded. (20)

The reference and citation lists of papers found to meet inclusion criteria will be scrutinised for further relevant papers. In addition, the corresponding authors will be contacted for full text versions of relevant abstracts, that are not available through the University subscription. Where authors have not responded within two weeks, the study will be excluded.

Selection process

The initial screening of all the titles/abstracts of the full search results, to determine whether papers meet the inclusion criteria, will be performed by the first reviewer (HKJ). The full search results will be sorted into one of three categories: 'include', 'exclude' and 'unsure'. Any issues in screening at this point will be discussed with the research team, to further develop the inclusion/exclusion criteria. The second reviewer (WO) will independently screen a randomly selected 10% of each of the included and excluded papers, and all of the 'unsure' papers. Any remaining contentious titles/abstracts will be re-assessed and independently screened by a third reviewer (GB).

All full-text versions of relevant titles/abstracts will then be retrieved and evaluated for eligibility by the first reviewer (HKJ). Where there is uncertainty around the eligibility of any papers, these will be discussed between two reviewers. If the disagreement persists, a third reviewer will make a final decision.

Data extraction and management

Search results will be exported into EndNote X9, where the data will be stored and managed. Duplicate results will be removed and numerical results of each stage of the systematic review will be illustrated in a PRISMA flow diagram. (21)

Data will be extracted by the first reviewer (HKJ) from a sample of the included studies, using a preliminary form. The data extraction form will then be revised as needed, following discussion with the research team, and the first reviewer (HKJ) will extract data from all included studies using the pilot-tested extraction form. The four proposed domains in the data extraction form are: (i) reference details (title, publication year, authors, journal); (ii) study details (aims, study setting, methods, participant characteristics); (iii) results and key findings; (iv) limitations (e.g. evidence of bias). If data are missing, the study authors will be contacted. Where authors have not responded within two weeks, the data will be excluded.

For peer review only - http://bmjopen.bmj.com/site/about/guidelines.xhtml

For peer review only

The second reviewer (WO) will independently extract data from 10% of the included studies. These data extraction forms will be compared between the first two reviewers, to assess for inter-rater reliability and ensure homogeneity.

Risk of bias (quality) assessment

The 2018 Mixed-Methods Assessment Tool (MMAT) will be used to assess the quality of individual studies, as it permits the appraisal of qualitative studies and mixed-methods studies, (22) both of which are in our inclusion criteria. The quality of each included study will be assessed independently by two reviewers. As quality assessment of qualitative sources is notoriously subjective, (23) studies of low methodological quality will still be included. We will however discuss low scores and their reasons in the final reporting, if a low-quality paper makes significant and unique contributions.

Data synthesis

Given that this review focuses predominantly on qualitative research findings, traditional methods of data synthesis found in aggregative reviews, such as meta-analysis, will not be appropriate. To account for and standardise data synthesis in systematic reviews which include a range of research designs, the UK ESRC Methods Programme has produced guidance on conducting 'narrative synthesis'. (24) A variation of this framework, adapted by Schofield and colleagues, (25) to synthesise data not focused on a particular intervention, will be utilised. A preliminary synthesis of the data, using the methods of 'thematic synthesis' developed by Thomas and Harden, will be undertaken to integrate themes and content that emerge from the included studies. Thematic synthesis has three stages: the coding of text 'line-by-line'; the development of 'descriptive themes'; and the generation of 'analytical themes'. (26) NVivo software will be used to undertake this qualitative data analysis. Relationships in the data will subsequently be explored, within and across the included studies. The strength of the evidence will then be assessed and the data will finally be synthesised to develop a theoretical model of physician approaches to conflict resolution in the adult intensive care unit. (24)

As we aim to synthesise qualitative data, the GRADE-CERQual approach has been chosen to assess confidence in the findings of this review, as it provides a systematic and transparent framework for assessing confidence in qualitative evidence synthesis. This is based on the consideration of four components: (i) methodological limitations, (ii) coherence, (iii) adequacy of data, and (iv) relevance. This will permit an assessment of whether the review findings are a reasonable representation of the phenomenon of interest. (27)

For peer review only - http://bmjopen.bmj.com/site/about/guidelines.xhtml

Patient and public involvement

There was no patient or public involvement in designing this protocol, although the findings will inform planned research with patients and healthcare professionals.

ETHICS AND DISSEMINATION

Ethical approval is not required for this review, as only published data will be included. We anticipate that the findings will be of interest to healthcare professionals working in intensive care units and individuals working in conflict resolution spheres (e.g. lawyers and mediators). The findings are also likely to be of interest to researchers in these fields, as well as those within the inter-disciplinary field of bioethics, given the ethically contentious nature of EOL decisions. We aim to disseminate findings both at academic conferences and through open-access publication in a peer-reviewed journal.

DISCUSSION

This systematic review will be the first, to our knowledge, to synthesise physician approaches to conflict resolution in EOL decisions in the AICU. Whilst previous reviews have explored the experiences of EOL decision-making for ICU healthcare professionals, or family satisfaction with EOL care in intensive care units; (7, 18) this will be the first to focus specifically on methods, employed by physicians, to resolve conflict. We hope that the findings of this review will therefore go on to inform educational curricula and training for ICU healthcare professionals, who may face conflict in EOL decision-making. It is also feasible that the findings may be transferable to other medical specialties, in which EOL conflicts may also arise, such as geriatrics and palliative care. Furthermore, we hope the findings will benefit individuals working in conflict resolution circles, as the review will evaluate the existing evidence to highlight both helpful and unhelpful methods. Additionally, by establishing what is currently known about physician approaches to resolving conflict, gaps in the evidence base can be identified, in order to guide future research.

There are potential limitations to this review. First, focussing on EOL disagreements may limit the identification of conflict resolution methods that are successfully employed in AICUs, to resolve other forms of conflict. For example, conflicts over pain management would not be included in this study but may contribute to discord between a patient's representatives and the responsible physician. However, given the intensity of EOL conflicts, if we are able to identify methods that physicians utilise to successfully resolve these conflicts through our review, these methods could potentially be employed to a certain degree in resolving other forms of conflicts (both within and outside the AICU). It is also a necessary restriction, to make the review more feasible.

For peer review only - http://bmjopen.bmj.com/site/about/guidelines.xhtml

Second, we focus solely on physicians' approaches to resolving conflicts (albeit taking stakeholders' perceptions of physician approaches into account). This may seem to overlook the role of nursing staff, who spend the most time at the patient's bedside, and therefore develop close relationships with both the patient and relatives. (7) It has been found that in critical care, nurses develop a sense of advocacy – that is, an obligation to support the patient's best interests. This may not always align with the physician's view and arguably contributes to the high prevalence of nurse-physician conflicts. (6) The relationship between nurses and patients is certainly of interest, as nursing staff may also offer strategies to resolve conflicts. However, as Flannery and colleagues reported, the responsibility for EOL decision-making ultimately lies with the physician, and we have therefore chosen to concentrate our review on physician approaches to resolving conflicts. (7) Moreover, this review does not consider external interventions e.g. legal advice, clinical ethics consultation and mediation. Although these methods have their own place and importance within conflict resolution spheres, we are particularly interested in the strategies that physicians employ in their day-to-day practice within the AICU, to address EOL disagreements when they arise. Considering all methods of EOL dispute resolution is beyond the scope of this systematic review, however they will be considered in a narrative review that is also being undertaken as part of the larger empirical bioethics study.

Finally, we have chosen not to apply methodological filters to identify qualitative research, due to the concern that methodological filters may result in loss of relevant studies. Instead, keywords have been chosen to identify qualitative research (as shown in Figure 1). These keywords were chosen based on previous systematic reviews and pilot searches. They have also been adapted to the keyword catalogues and indexing vocabulary in each database. Following a pilot search on MEDLINE, the keywords retrieved the same studies as a qualitative research methodological filter, and more. Using these keywords successfully identified studies of interest, which were known to the research team prior to commencing the review. It is still possible that a relevant study may be missed by the search strategy. We hope to circumvent this, by hand-searching the reference lists and citation lists of included studies. Additionally, how to best assess the quality of qualitative studies is widely debated. Due to our inclusion of the qualitative data from mixed methods studies, using the MMAT will allow us to use one tool to assess the quality of both purely qualitative and mixed methods studies. The MMAT has fewer criteria than alternatives (e.g. the Critical Appraisal Skills Programme Qualitative 2018 checklist (28)) and may therefore not provide as thorough an assessment of quality. However, as we do not intend to exclude studies of low methodological quality, the MMAT will still allow us to factor low scores into our overall analysis of the data.

Reporting

For peer review only - http://bmjopen.bmj.com/site/about/guidelines.xhtml

The review will be reported in accordance with the PRISMA statement. (21) Any important amendments to the protocol will be documented on PROSPERO and in the final report.

Acknowledgements

The authors would like to thank Sarah Herring, for her help in refining the search strategy for this review and navigating online databases, and William Orchard, for his input as the second reviewer.

Authors' Contributions

HKJ, GB and RH conceived of the review, developed the protocol and developed the search strategy. HKJ drafted the manuscript and is the guarantor of this review. All authors revised and edited the draft manuscript and approved the final version.

Funding

The work is based at the University of Bristol, as part of the 'Balancing Best Interests in Health Care, Ethics and Law (BABEL)' Collaborative Award. This research was funded in whole, or in part, by the Wellcome Trust Grant no. 209841/Z/17/Z. The funders had no role in the preparation of this manuscript or the decision to submit for publication. For the purpose of Open Access, the author has applied a CC BY public copyright licence to any Author Accepted Manuscript version arising from this submission.

Competing Interests

None declared.

Patient Consent

Not required.

REFERENCES

1. Knickle K, McNaughton N, Downar J. Beyond winning: mediation, conflict resolution, and non-rational sources of conflict in the ICU. Critical Care. 2012;16:308.

For peer review only - http://bmjopen.bmj.com/site/about/guidelines.xhtml

2. Choong K, Cupido C, Nelson E, Arnold DM, Burns K, Cook D, et al. A framework for resolving disagreement during end of life care in the critical care unit. Clin Invest Med. 2010;33(4):240-53.
3. Re Y [2018] UKSC 46.
4. Meller S, Barclay S. Mediation: an approach to intractable disputes between parents and paediatricians. Archives of Disease in Childhood. 2011;96:619-21.
5. Johal HK, Danbury C. Conflict before the courtroom: challenging cognitive biases in critical decision-making. Journal of Medical Ethics. 2020:medethics-2020-106177.
6. Azoulay E, Timsit JF, Sprung CL. Prevalence and factors of intensive care unit conflicts: the conflicus study. Am J Respir Crit Care Med. 2009;180:853-60.
7. Flannery L, Ramjan LM, Peters K. End-of-life decisions in the intensive care unit (ICU) - exploring the experiences of ICU nurses and doctors - a critical literature review. Aust Crit Care. 2016;29(2):97-103.
8. Austin L, Huxtable R. Resolving disagreements about the care of critically ill children: Evaluating existing processes and setting the research agenda. Parental rights, best interests and significant harms: Medical decision-making on behalf of children post-Great Ormond Street Hospital v Yates: Hart Publishing; 2019.
9. Mehter HM, McCannon JB, Clark JA, Wiener RS. Physician approaches to conflict with families surrounding end-of-life decision-making in the intensive care unit. A qualitative study. Ann Am Thorac Soc. 2018;15(2):241-9.
10. Braunack-Mayer A. What makes a problem an ethical problem? An empirical perspective on the nature of ethical problems in general practice. Journal of Medical Ethics. 2001;27:98-103.
11. Guyatt G, Cairns J, Churchill D, Cook D, Haynes B, Hirsh J, et al. Evidence-based medicine: A new approach to teaching the practice of medicine. JAMA. 1992;268(17):2420-5.
12. Huxtable R, Ives J. Mapping, framing, shaping: a framework for empirical bioethics research projects. BMC Medical Ethics. 2019;20(1):1-8.
13. Ives J. A method of reflexive balancing in a pragmatic, interdisciplinary and reflexive bioethics. Bioethics. 2014;28(6):302-12.
14. Olmstead JA, Dahnke MD. The need for an effective process to resolve conflicts over medical futility: A case study and analysis. Critical Care Nurse. 2016;36(6):13-23.
15. Shamseer L, Moher D, Clarke M, Ghersi D, Liberati A, Petticrew M, et al. Preferred reporting items for systematic review and meta-analysis protocols (PRISMA-P) 2015 statement. Syst Rev. 2015;4(1).
16. Strech D, Synofzik M, Marckmann G. Systematic reviews of empirical bioethics. Journal of Medical Ethics. 2008;34:472-7.
17. Taylor H. What are 'best interests'? A critical evaluation of 'best interests' decision-making in clinical practice. Medical Law Review. 2016;24(2):176-205.
18. Schram A, Hougham G, Meltzer D, Ruhnke G. Palliative care in critical care settings: A systematic review of communication-based competencies essential for patient and family satisfaction. American Journal of Hospice & Palliative Medicine. 2017;34(9):887-95.
19. Lefebvre C, Manheimer E, Glanville J. Searching for studies. In: Higgins J, Greene S, editors. Cochrane handbook for systematic reviews of interventions, version 5 2008.
20. Mahood Q, Eerd DV, Irvin E. Searching for grey literature for systematic reviews: challenges and benefits. Research Synthesis Methods. 2013;5(3):221-34.
21. Moher D, Liberati A, Tetzlaff J, Altman D. Preferred reporting items for systematic Reviews and meta-analyses: The PRISMA statement. Open Med. 2009;3(3):123-30.
22. Hong Q, Pluye P, Fàbregues S, Bartlett G, Boardman F, Cargo M, et al. Mixed Methods Appraisal Tool (MMAT), version 2018. Registration of Copyright (#1148552), Canadian Intellectual Property Office, Industry Canada.
23. Dixon-Woods M, Sutton A, Shaw R, Miller T, Smith J, Young B, et al. Appraising qualitative research for inclusion in systematic reviews: a quantitative and qualitative comparison of three methods. J Health Serv Res Policy. 2007;12(1):42-7.
24. Popay J, Roberts H, Sowden A, Petticrew M, Arai L, Rodgers M, et al. Guidance on the conduct of narrative synthesis in systematic reviews. Product from the ESRC Methods Programme. 2006;1:b92.

For peer review only - http://bmjopen.bmj.com/site/about/guidelines.xhtml

25. Schofield G, Brangan E, Dittborn M, Huxtable R, Selman L. Real-world ethics in palliative care: protocol for a systematic review of the ethical challenges reported by specialist palliative care practitioners in their clinical practice. BMJ Open. 2019;9:e028480:doi:10.1136/bmjopen-2018-028480.
26. Thomas J, Harden A. Methods for the thematic synthesis of qualitative research in systematic reviews. BMC Medical Research Methodology. 2008;8(45).
27. Lewin S, Booth A, Glenton C, Munthe-Kaas H, Rashidian A, Wainwright M, et al. Applying GRADE-CERQual to qualitative evidence synthesis findings: introduction to the series. Implementation Science. 2018;13(2):1-10.
28. Programme CAS. CASP Qualitative Checklist 2018 [Available from: https://casp-uk.net/wp-content/uploads/2018/01/CASP-Qualitative-Checklist-2018.pdf.

For peer review only

For peer review only - http://bmjopen.bmj.com/site/about/guidelines.xhtml

MEDLINE Search Strategy

1. Terminal Care/
2. Decision Making/
3. Exp Refusal to Treat/
4. end of life.mp.
5. best interest*.mp.
6. withdraw*.mp.
7. withhold*.mp.
8. decision*.mp.
9. resol*.mp.
10. consent.mp.
11. refusal*.mp
12. **1 or 2 or 3 or 4 or 5 or 6 or 7 or 8 or 9 or 10 or 11**
13. Intensive Care Units/
14. critical care*.mp.
15. intensive care*.mp.
16. adult ICU.mp.
17. **13 or 14 or 15 or 16**
18. Dissent and Disputes/
19. conflict*.mp.
20. dispute*.mp.
21. disagreement*.mp
22. dissent*.mp
23. **18 or 19 or 20 or 21 or 22**
24. Physicians/
25. physician*.mp.
26. doctor*.mp.
27. nurse*.mp.
28. healthcare professional*.mp.
29. famil*.mp.
30. relative*.mp.
31. therap*.mp.
32. surrogate*.mp.
33. prox*.mp.
34. patient*.mp.
35. **24 or 25 or 26 or 27 or 28 or 29 or 30 or 31 or 32 or 33 or 34**
36. qualitative.mp.
37. interview*.mp.
38. focus group*
39. survey*.mp.
40. questionnaire*.mp.
41. empirical*.mp.
42. framework*.mp.
43. **36 or 37 or 38 or 39 or 40 or 41 or 42**
44. **12 and 17 and 23 and 35 and 43**

For peer review only

Reporting checklist for protocol of a systematic review and meta analysis.

Based on the PRISMA-P guidelines.

		Reporting Item	Page Number
**Title**			
Identification	#1a	Identify the report as a protocol of a systematic review	5
Update	#1b	If the protocol is for an update of a previous systematic review, identify as such	n/a
**Registration**			
	#2	If registered, provide the name of the registry (such as PROSPERO) and registration number	1
**Authors**			
Contact	#3a	Provide name, institutional affiliation, e-mail address of all protocol authors; provide physical mailing address of corresponding author	1
Contribution	#3b	Describe contributions of protocol authors and identify the guarantor of the review	14
**Amendments**			
	#4	If the protocol represents an amendment of a previously	13

For peer review only

		completed or published protocol, identify as such and list changes; otherwise, state plan for documenting important protocol amendments	
**Support**			
Sources	#5a	Indicate sources of financial or other support for the review	14
Sponsor	#5b	Provide name for the review funder and / or sponsor	14
Role of sponsor or funder	#5c	Describe roles of funder(s), sponsor(s), and / or institution(s), if any, in developing the protocol	14
**Introduction**			
Rationale	#6	Describe the rationale for the review in the context of what is already known	4
Objectives	#7	Provide an explicit statement of the question(s) the review will address with reference to participants, interventions, comparators, and outcomes (PICO)	5
**Methods**			
Eligibility criteria	#8	Specify the study characteristics (such as PICO, study design, setting, time frame) and report characteristics (such as years considered, language, publication status) to be used as criteria for eligibility for the review	5
Information sources	#9	Describe all intended information sources (such as electronic databases, contact with study authors, trial registers or other grey literature sources) with planned dates of coverage	9

For peer review only

Search strategy	#10	Present draft of search strategy to be used for at least one electronic database, including planned limits, such that it could be repeated	8
Study records - data management	#11a	Describe the mechanism(s) that will be used to manage records and data throughout the review	10
Study records - selection process	#11b	State the process that will be used for selecting studies (such as two independent reviewers) through each phase of the review (that is, screening, eligibility and inclusion in meta-analysis)	10
Study records - data collection process	#11c	Describe planned method of extracting data from reports (such as piloting forms, done independently, in duplicate), any processes for obtaining and confirming data from investigators	10
Data items	#12	List and define all variables for which data will be sought (such as PICO items, funding sources), any pre-planned data assumptions and simplifications	10
Outcomes and prioritization	#13	List and define all outcomes for which data will be sought, including prioritization of main and additional outcomes, with rationale	n/a
Risk of bias in individual studies	#14	Describe anticipated methods for assessing risk of bias of individual studies, including whether this will be done at the outcome or study level, or both; state how this information will be used in data synthesis	11
Data synthesis	#15a	Describe criteria under which study data will be quantitatively	n/a

For peer review only

		synthesised	
Data synthesis	#15b	If data are appropriate for quantitative synthesis, describe planned summary measures, methods of handling data and methods of combining data from studies, including any planned exploration of consistency (such as I2, Kendall's τ)	n/a
Data synthesis	#15c	Describe any proposed additional analyses (such as sensitivity or subgroup analyses, meta-regression)	n/a
Data synthesis	#15d	If quantitative synthesis is not appropriate, describe the type of summary planned	11
Meta-bias(es)	#16	Specify any planned assessment of meta-bias(es) (such as publication bias across studies, selective reporting within studies)	11
Confidence in cumulative evidence	#17	Describe how the strength of the body of evidence will be assessed (such as GRADE)	11

The PRISMA-P elaboration and explanation paper is distributed under the terms of the Creative Commons Attribution License CC-BY. This checklist was completed on 13. September 2021 using https://www.goodreports.org/, a tool made by the EQUATOR Network in collaboration with Penelope.ai

For peer review only

BMJ Open

Protocol for a Systematic Review of Qualitative Research Exploring Physician Approaches to Conflict Resolution in End-of-Life Decisions in the Adult Intensive Care Unit

Journal:	*BMJ Open*
Manuscript ID	bmjopen-2021-057387.R1
Article Type:	Protocol
Date Submitted by the Author:	31-Mar-2022
Complete List of Authors:	Johal, Harleen; University of Bristol Centre for Ethics in Medicine, Bristol Medical School<br>Birchley, Giles; University of Bristol Centre for Ethics in Medicine<br>Huxtable, Richard; University of Bristol Centre for Ethics in Medicine
**Primary Subject Heading**:	Intensive care
Secondary Subject Heading:	Qualitative research, Ethics, Communication, Palliative care
Keywords:	INTENSIVE & CRITICAL CARE, MEDICAL ETHICS, Adult intensive & critical care < INTENSIVE & CRITICAL CARE, Adult palliative care < PALLIATIVE CARE, PALLIATIVE CARE, Adult intensive & critical care < ANAESTHETICS

SCHOLARONE™
Manuscripts

Harleen Kaur Johal, Giles Birchley & Richard Huxtable

Protocol for a Systematic Review of Qualitative Research Exploring Physician Approaches to Conflict Resolution in End-of-Life Decisions in the Adult Intensive Care Unit

Authors

- Harleen Kaur Johal (***Corresponding Author***)
  Centre for Ethics in Medicine, Population Health Sciences, Bristol Medical School, University of Bristol
  (E-mail: harleen.johal@bristol.ac.uk)
- Giles Birchley
  Centre for Ethics in Medicine, Population Health Sciences, Bristol Medical School, University of Bristol
  (E-mail: giles.birchley@bristol.ac.uk)
- Richard Huxtable
  Centre for Ethics in Medicine, Population Health Sciences, Bristol Medical School, University of Bristol
  (E-mail: r.huxtable@bristol.ac.uk)

Registration

PROSPERO CRD42021193769

Word Count

3643 words

For peer review only

For peer review only - http://bmjopen.bmj.com/site/about/guidelines.xhtml

Harleen Kaur Johal, Giles Birchley & Richard Huxtable

ABSTRACT

Introduction

Conflict is unfortunately well-documented in the adult intensive care unit (AICU). In the context of end-of-life (EOL) decision-making (i.e., the withdrawal or withholding of life-sustaining treatment), conflict commonly occurs when a consensus cannot be reached between the healthcare team and the patient's family on the "best interests" of the critically ill, incapacitated patient. Whilst existing literature has identified potential methods for conflict resolution, it is less clear how these approaches are perceived and utilised by stakeholders in the EOL decision-making process. We aim to explore this by systematically reviewing and synthesising the published evidence, which addresses the following research question: what does existing qualitative research reveal about physician approaches to addressing conflict arising in EOL decisions in the AICU?

Methods and Analysis

Peer-reviewed qualitative studies (retrieved from MEDLINE, Project Muse, Scopus, EMBASE, Web of Science, PsycINFO, CINAHL and LILACS) examining conflict and dispute resolution in the context of EOL decisions in the AICU setting will be included. Two reviewers will independently screen either all or a randomly selected sample of studies, with a third reviewer independently screening studies of uncertain eligibility. The 'thematic synthesis' approach will be employed to analyse the resulting data (Thomas & Harden, 2008). The quality of included papers will be assessed using the 2018 Mixed-Methods Assessment Tool (MMAT). The GRADE-CERQual approach will be utilised to assess our confidence in the findings.

Ethics and Dissemination

Ethical approval is not required for this review, as only published data will be included. We anticipate that the findings will be of interest to healthcare professionals working in AICUs and individuals working in bioethics, given the ethically contentious nature of EOL decisions. The findings will be disseminated at both academic conferences and through open-access publication in a peer-reviewed journal. The protocol has been registered on the PROSPERO database (CRD42021193769).

300 words

For peer review only

For peer review only - http://bmjopen.bmj.com/site/about/guidelines.xhtml

Harleen Kaur Johal, Giles Birchley & Richard Huxtable

ARTICLE SUMMARY

Strengths and Limitations of This Study

- This systematic review has clear scope, with predefined inclusion and exclusion criteria.
- The reviewers will identify additional articles of interest by hand-searching the reference and citation lists of included papers.
- The search strategy benefits from utilising a range of bibliographic databases, including those which index both philosophical and clinical research.
- The exclusion of other healthcare professionals' approaches (such as nurses) to resolving conflict may result in effective approaches not being identified.
- By focusing on qualitative data, rather than theoretical or normative research, the findings of this review will better represent the reality and feasibility of conflict resolution in clinical practice.

For peer review only

For peer review only - http://bmjopen.bmj.com/site/about/guidelines.xhtml

INTRODUCTION

Conflict is unfortunately well-documented in the adult intensive care unit (AICU). In the context of end-of-life (EOL) decision-making (i.e. the withdrawal or withholding of life-sustaining treatment), it commonly occurs when a consensus cannot be reached on the best interests of the critically ill, incapacitated patient. (1) Families, as surrogate decision-makers, may favour preservation of life, whilst physicians may be reluctant to provide life-sustaining treatment, which is potentially inappropriate. (2) The decision-making process for healthcare professionals (HCPs) is informed by their understanding of the law and professional guidance. In England and Wales, when there is an agreement on the best interests of a patient, there is no need to make an application to the court to withdraw or withhold life-sustaining treatment. (3) Involvement of the courts is however necessitated where disagreement between the conflicting parties has become intractable. This adversarial process exacerbates distress amongst HCPs and families, potentially leading to breakdowns in therapeutic relationships. (4) This arguably justifies the utilisation of alternative methods of conflict resolution, before a dispute becomes sufficiently entrenched to require legal intervention. (1, 5)

There is an extensive body of literature recognising the incidence of conflict in adult ICUs. The 'Conflicus' study surveyed the experiences of 7498 ICU staff members in 323 ICUs in 24 countries. Nurse-physician conflicts were most common (32.6%) and staff-relative conflicts accounted for 26.2% of perceived conflicts. Lack of psychological support and problems with the decision-making process were identified as causes of conflict in EOL care. (6) Indeed, in a critical literature review exploring doctors' and nurses' EOL decision-making process, Flannery and colleagues suggested that more comprehensive and standardised approaches are needed to support all HCPs in making these difficult decisions. (7) One could extend this suggestion into a need for more guidance, to support HCPs in the resolution of disputes when they occur. Whilst academic literature has identified potential routes for dispute resolution, for example, clinical ethics committees and mediation, (8) it is less clear how these approaches are perceived and utilised by stakeholders in the EOL decision-making process, such as healthcare professionals, patients and surrogate decision-makers. (9) It has also been observed in other areas of healthcare that theoretical discussion of ethical challenges is not necessarily representative of the ethical issues that arise in real clinical practice. (10) Further consideration should therefore be given to how these stakeholders actually approach conflict and how they perceive strategies for conflict resolution.

We seek to explore this by systematically reviewing and synthesising the published qualitative evidence on physician approaches to conflict resolution in EOL decisions in the AICU. A coherent and cohesive understanding of current approaches to conflict resolution will provide a starting point for an evidence-base, from which healthcare professionals can be trained in how to approach conflict. (11) The rationale for this review is further supported by recent developments in the field of bioethics, where the value of

For peer review only - http://bmjopen.bmj.com/site/about/guidelines.xhtml

empirical evidence in evaluating normative claims has been increasingly recognised. (12) Hence, in order to address the question of how conflict should be resolved, we must first consider the context of conflict and how physicians attempt to resolve it, so the resulting ethical analysis is informed by and more relevant to clinical practice. (13)

This systematic review shall lay the foundations of a larger empirical bioethics study, in which disagreements between healthcare professionals and patient representatives will be explored. Ultimately, the development of guidelines to support all healthcare professionals, patients and families when conflict arises, will aim to improve their experience during an ethically contentious and emotionally strenuous time. (14)

Aims

This systematic review protocol has been guided by the Preferred Reporting Items for Systematic Reviews and Meta-Analyses Protocols (PRISMA-P) checklist (see online supplementary file), (15) and the review will aim to address the following research question: what does existing qualitative research reveal about physician approaches to addressing conflict arising in EOL decisions (specifically, the withdrawal and withholding of life-sustaining treatments) in the AICU? The standard "Participants, Interventions, Comparators and Outcomes" (PICO) system used in reviews of clinical studies has been modified here, as it is less suited to qualitative evidence synthesis, by using Strech and colleagues' "Methodology, Issue, Participants" (MIP) system. (16)

METHODS AND ANALYSIS

Eligibility criteria

The purpose of this review is to identify studies in which physicians have described their strategies for approaching and resolving conflict around EOL decisions in the AICU. The inclusion and exclusion criteria, following Strech and colleagues' MIP system, are shown in table 1. Peer-reviewed qualitative studies examining conflict resolution in the context of EOL decisions in the adult intensive care setting will therefore be included.

Studies identifying factors, which contribute to the development of conflict in AICU around EOL decisions, will not be included. Identification of these factors is important in the development of conflict resolution strategies, however this would constitute a separate systematic review. Studies relating to conflict around EOL decisions for critically ill children and neonates will also be excluded. Whilst there may be similar circumstances to those which present in an adult intensive care environment, where the patient is incapacitated and a decision must therefore be made in their best interests, there are legal and

For peer review only - http://bmjopen.bmj.com/site/about/guidelines.xhtml

For peer review only

clinical differences between adults and children. The role of the "family and others" is notably different in best interests decision-making processes for adults,(17) as compared to children; and the resulting conflicts may be different in nature. Whilst conflict resolution strategies in AICU could be applied in PICU and vice versa, it is beyond the scope of this review to consider two clinical contexts, with potentially crucial differences, and two groups of stakeholders with differing roles.

Table 1: Inclusion and Exclusion Criteria

	**Inclusion Criteria**	**Exclusion Criteria**
**Context**	Critical/intensive care settings where EOL decisions are made for adults will be included.<br>The terms 'critical' and 'intensive' are often used interchangeably for clinical settings in which patients are seriously ill and/or require some form of life-sustaining treatment.	Clinical settings, in which EOL decision are made for children or neonates will be excluded.<br>Non-critical/intensive care settings will be excluded.<br>Other specialities or settings, in which EOL decisions are made (e.g. community palliative care), will be excluded.
**Methodologies**	Qualitative studies examining conflict resolution in the AICU (e.g. interviews and focus groups).<br>Mixed-methods studies (e.g. surveys with open questions) will also be included for consideration of the qualitative evidence presented.	Non-empirical studies examining conflict resolution. This may include normative and theoretical literature. These have been excluded as we are interested in understanding the lived reality of conflict resolution in clinical practice.<br>Quantitative studies examining conflict resolution in the AICU. These have been excluded as we are interested in deeper exploration of experiences in the AICU, which is not possible using quantitative methods.
**Issues**	Qualitative studies exploring conflict resolution strategies within	Literature that does not explore conflict, dispute, disagreement,

For peer review only - http://bmjopen.bmj.com/site/about/guidelines.xhtml

	the AICU, around EOL decisions, will be included. EOL decisions are here defined as decisions relating to the withdrawal or withholding of life-sustaining treatment (e.g., dialysis, clinically-assisted nutrition and hydration, artificial ventilation) or decisions relating to cardiopulmonary resuscitation. Conflict is here defined as a failure to reach an agreement on whether life-sustaining treatment should be withdrawn or withheld. The terms ‘conflict’, ‘dispute’, ‘disagreement’, ‘dissent’ and ‘refusal’ are often used interchangeably in the existing literature, and we are therefore interested in studies which explore any of these issues.	dissent, and refusal will be excluded. Literature that does not explore withdrawal or withholding of life-sustaining treatment will be excluded. Empirical studies examining factors, which contribute to conflict, will be excluded. Whilst the identification of factors which cause conflict is fundamental to the development of conflict resolution strategies, it is beyond the scope of this systematic review.
**Participants**	Studies which explore physician approaches to conflict resolution will be included. Studies which explore other stakeholders’ perceptions of physician approaches to conflict resolution will also be included. Stakeholders are broadly-defined in three categories: (i) healthcare professionals (e.g. physicians/doctors, nurses and therapists), (ii) adult patients and (iii) patient representatives (e.g. families, spouses, relatives and other surrogate decision-makers).	Literature that does not encompass discussion of stakeholders’ (as defined in the inclusion criteria) approaches to conflict in EOL decisions in the AICU, will be excluded. Literature that discusses non-physician led approaches to conflict resolution, e.g., clinical ethics consultation or independent mediation, will be excluded.

For peer review only

For peer review only - http://bmjopen.bmj.com/site/about/guidelines.xhtml

**Timeframe**	Any studies published after 2000 will be included, as critical care is a rapidly developing field.	Studies published before 2000 will be excluded, as they are less likely to be relevant to current clinical practice.
**Types of Publications**	Peer-reviewed journal publications of empirical research.<br>Publications in English.<br>International publications will be included.	Unpublished and grey literature, theses and dissertations, and any published sourced that do not contain empirical studies will be excluded.<br>Publications not in English will be excluded.<br>Conference abstracts will be excluded.<br>Review articles relating to the research question will be used for the identification of empirical studies only.<br>Study authors will be contacted if we are unable to obtain the full text through the university subscription. If the study authors do not respond to this request within two weeks, the study will be excluded.

For peer review only

Search strategy

Scoping searches were conducted, using MEDLINE Subject Headings (MeSH) and truncations of keywords obtained from a previous systematic review of palliative care in the AICU. (18) The search strategy was further refined following consultation with a librarian, by checking the MESH database to identify relevant concepts and select appropriate terms.[1] Descriptors and synonyms were both used, in addition to truncations and abbreviations of terms, to retrieve all related variants. (19) The MEDLINE

[1] While some of the subject heading terms may appear contradictory, we have included all terms that relate to the withdrawal or withholding of treatment, regardless of whether this is done as part of a research protocol (e.g., MeSH term "Withholding Treatment") or whether treatment is withheld by the professional against the patient's or patient's representatives' wishes (e.g., MeSH term "Refusal to Treat"). This is to identify any potential conflicts that may arise when making decisions about life-sustaining treatment. Many thanks to Dr Dina Vrkić for highlighting this.

For peer review only - http://bmjopen.bmj.com/site/about/guidelines.xhtml

search strategy was then amended for other databases as needed, including adjustments to subject headings (see online supplementary file).

Scoping searches for the study began in March 2021, and the planned end date for the systematic review is July 2022.

Information sources

We will use a combination of general bibliographic (Web of Science, Scopus), subject-specific bibliographic (MEDLINE, EMBASE, PsycINFO, CINAHL, and LILACS), and full-text (Project MUSE) databases. These databases have been chosen as they are known to be directly relevant to studies conducted in medicine, social sciences and bioethics. We have imposed a restriction on publication dates, as critical care is a rapidly developing field and studies pre-2000 are likely to be outdated. We will also only include papers in English. Due to the resource-intensive nature of reviewing grey literature, it will also be excluded. (20)

The reference and citation lists of papers found to meet inclusion criteria will be scrutinised for further relevant papers. In addition, the corresponding authors will be contacted for full text versions of relevant abstracts, that are not available through the University subscription. Where authors have not responded within two weeks, the study will be excluded.

Selection process

The initial screening of all the titles/abstracts of the full search results, to determine whether papers meet the inclusion criteria, will be performed by the first reviewer (HKJ). The full search results will be sorted into one of three categories: 'include', 'exclude' and 'unsure'. Any issues in screening at this point will be discussed with the research team, to further develop the inclusion/exclusion criteria. The second reviewer (WO) will independently screen a randomly selected 10% of each of the included and excluded papers, and all of the 'unsure' papers. Any remaining contentious titles/abstracts will be re-assessed and independently screened by a third reviewer (GB).

All full-text versions of relevant titles/abstracts will then be retrieved and evaluated for eligibility by the first reviewer (HKJ). Where there is uncertainty around the eligibility of any papers, these will be discussed between two reviewers. If the disagreement persists, a third reviewer will make a final decision.

Data extraction and management

For peer review only - http://bmjopen.bmj.com/site/about/guidelines.xhtml

Search results will be exported into EndNote X9, where the data will be stored and managed. Duplicate results will be removed and numerical results of each stage of the systematic review will be illustrated in a PRISMA flow diagram. (21)

Data will be extracted by the first reviewer (HKJ) from a sample of the included studies, using a preliminary form. The data extraction form will then be revised as needed, following discussion with the research team, and the first reviewer (HKJ) will extract data from all included studies using the pilot-tested extraction form. The four proposed domains in the data extraction form are: (i) reference details (title, publication year, authors, journal); (ii) study details (aims, study setting, methods, participant characteristics); (iii) results and key findings; (iv) limitations (e.g. evidence of bias). If data are missing, the study authors will be contacted. Where authors have not responded within two weeks, the data will be excluded.

The second reviewer (WO) will independently extract data from 10% of the included studies. These data extraction forms will be compared between the first two reviewers, to assess for inter-rater reliability and ensure homogeneity.

Risk of bias (quality) assessment

The 2018 Mixed-Methods Assessment Tool (MMAT) will be used to assess the quality of individual studies, as it permits the appraisal of qualitative studies and mixed-methods studies, (22) both of which are in our inclusion criteria. The quality of each included study will be assessed independently by two reviewers. As quality assessment of qualitative sources is notoriously subjective, (23) studies of low methodological quality will still be included. We will however discuss low scores and their reasons in the final reporting, if a low-quality paper makes significant and unique contributions.

Data synthesis

Given that this review focuses predominantly on qualitative research findings, traditional methods of data synthesis found in aggregative reviews, such as meta-analysis, will not be appropriate. To account for and standardise data synthesis in systematic reviews which include a range of research designs, the UK ESRC Methods Programme has produced guidance on conducting 'narrative synthesis'. (24) A variation of this framework, adapted by Schofield and colleagues, (25) to synthesise data not focused on a particular intervention, will be utilised. A preliminary synthesis of the data, using the methods of 'thematic synthesis' developed by Thomas and Harden, will be undertaken to integrate themes and content that emerge from the included studies. Thematic synthesis has three stages: the coding of text 'line-by-line'; the development of 'descriptive themes'; and the generation of 'analytical themes'. (26)

For peer review only - http://bmjopen.bmj.com/site/about/guidelines.xhtml

NVivo software will be used to undertake this qualitative data analysis. Relationships in the data will subsequently be explored, within and across the included studies. The strength of the evidence will then be assessed and the data will finally be synthesised to develop a theoretical model of physician approaches to conflict resolution in the adult intensive care unit. (24)

As we aim to synthesise qualitative data, the GRADE-CERQual approach has been chosen to assess confidence in the findings of this review, as it provides a systematic and transparent framework for assessing confidence in qualitative evidence synthesis. This is based on the consideration of four components: (i) methodological limitations, (ii) coherence, (iii) adequacy of data, and (iv) relevance. This will permit an assessment of whether the review findings are a reasonable representation of the phenomenon of interest. (27)

Patient and public involvement

There was no patient or public involvement in designing this protocol, although the findings will inform planned research with patients and healthcare professionals.

ETHICS AND DISSEMINATION

Ethical approval is not required for this review, as only published data will be included. We anticipate that the findings will be of interest to healthcare professionals working in intensive care units and individuals working in conflict resolution spheres (e.g., lawyers and mediators). The findings are also likely to be of interest to researchers in these fields, as well as those within the inter-disciplinary field of bioethics, given the ethically contentious nature of EOL decisions. We aim to disseminate findings both at academic conferences and through open-access publication in a peer-reviewed journal.

DISCUSSION

This systematic review will be the first, to our knowledge, to synthesise physician approaches to conflict resolution in EOL decisions in the AICU. Whilst previous reviews have explored the experiences of EOL decision-making for ICU healthcare professionals, or family satisfaction with EOL care in intensive care units, (7, 18) this will be the first to focus specifically on methods, employed by physicians, to resolve conflict. We hope that the findings of this review will therefore go on to inform educational curricula and training for ICU healthcare professionals, who may face conflict in EOL decision-making. It is also feasible that the findings may be transferable to other medical specialties, in which EOL conflicts may also arise, such as geriatrics and palliative care. Furthermore, we hope the

For peer review only - http://bmjopen.bmj.com/site/about/guidelines.xhtml

findings will benefit individuals working in conflict resolution circles, as the review will evaluate the existing evidence to highlight both helpful and unhelpful methods. Additionally, by establishing what is currently known about physician approaches to resolving conflict, gaps in the evidence base can be identified, in order to guide future research.

There are potential limitations to this review. First, focussing on EOL disagreements may limit the identification of conflict resolution methods that are successfully employed in AICUs, to resolve other forms of conflict. For example, conflicts over pain management would not be included in this study but may contribute to discord between a patient's representatives and the responsible physician. However, given the intensity of EOL conflicts, if we are able to identify methods that physicians utilise to successfully resolve these conflicts through our review, these methods could potentially be employed to a certain degree in resolving other forms of conflicts (both within and outside the AICU). It is also a necessary restriction, to make the review more feasible.

Second, we focus solely on physicians' approaches to resolving conflicts (albeit taking stakeholders' perceptions of physician approaches into account). This may seem to overlook the role of nursing staff, who spend the most time at the patient's bedside, and therefore develop close relationships with both the patient and relatives. (7) It has been found that in critical care, nurses develop a sense of advocacy – that is, an obligation to support the patient's best interests. This may not always align with the physician's view and arguably contributes to the high prevalence of nurse-physician conflicts. (6) The relationship between nurses and patients is certainly of interest, as nursing staff may also offer strategies to resolve conflicts. However, as Flannery and colleagues reported, the responsibility for EOL decision-making ultimately lies with the physician, and we have therefore chosen to concentrate our review on physician approaches to resolving conflicts. (7) Moreover, this review does not consider external interventions e.g. legal advice, clinical ethics consultation and mediation. Although these methods have their own place and importance within conflict resolution spheres, we are particularly interested in the strategies that physicians employ in their day-to-day practice within the AICU, to address EOL disagreements when they arise. Considering all methods of EOL dispute resolution is beyond the scope of this systematic review, however they will be considered in a narrative review that is also being undertaken as part of the larger empirical bioethics study.

Finally, we have chosen not to apply methodological filters to identify qualitative research, due to the concern that methodological filters may result in loss of relevant studies. Instead, keywords have been chosen to identify qualitative research (see online supplementary file). These keywords were chosen based on previous systematic reviews and pilot searches. They have also been adapted to the keyword catalogues and indexing vocabulary in each database. Following a pilot search on MEDLINE, the keywords retrieved the same studies as a qualitative research methodological filter, and more. Using these keywords successfully identified studies of interest, which were known to the research team prior

For peer review only - http://bmjopen.bmj.com/site/about/guidelines.xhtml

to commencing the review. It is still possible that a relevant study may be missed by the search strategy. We hope to circumvent this, by hand-searching the reference lists and citation lists of included studies. Additionally, how to best assess the quality of qualitative studies is widely debated. Due to our inclusion of the qualitative data from mixed methods studies, using the MMAT will allow us to use one tool to assess the quality of both purely qualitative and mixed methods studies. The MMAT has fewer criteria than alternatives (e.g. the Critical Appraisal Skills Programme Qualitative 2018 checklist (28)) and may therefore not provide as thorough an assessment of quality. However, as we do not intend to exclude studies of low methodological quality, the MMAT will still allow us to factor low scores into our overall analysis of the data.

Reporting

The review will be reported in accordance with the PRISMA statement. (21) Any important amendments to the protocol will be documented on PROSPERO and in the final report.

Acknowledgements

The authors would like to thank Sarah Herring, for her help in refining the search strategy for this review and navigating online databases; William Orchard, for his input as the second reviewer; and the reviewers of this manuscript for their helpful suggestions.

Authors' Contributions

HKJ, GB and RH conceived of the review, developed the protocol and developed the search strategy. HKJ drafted the manuscript and is the guarantor of this review. All authors revised and edited the draft manuscript and approved the final version.

Funding

The work is based at the University of Bristol, as part of the 'Balancing Best Interests in Health Care, Ethics and Law (BABEL)' Collaborative Award. This research was funded in whole, or in part, by the Wellcome Trust Grant no. 209841/Z/17/Z. The funders had no role in the preparation of this manuscript or the decision to submit for publication. For the purpose of Open Access, the author has applied a CC BY public copyright licence to any Author Accepted Manuscript version arising from this submission.

For peer review only - http://bmjopen.bmj.com/site/about/guidelines.xhtml

Competing Interests

None declared.

Patient Consent

Not required.

Figure Legends

Table 1: Inclusion and Exclusion Criteria

REFERENCES

1. Knickle K, McNaughton N, Downar J. Beyond winning: mediation, conflict resolution, and non-rational sources of conflict in the ICU. Critical Care. 2012;16:308.
2. Choong K, Cupido C, Nelson E, Arnold DM, Burns K, Cook D, et al. A framework for resolving disagreement during end of life care in the critical care unit. Clin Invest Med. 2010;33(4):240-53.
3. Re Y [2018] UKSC 46.
4. Meller S, Barclay S. Mediation: an approach to intractable disputes between parents and paediatricians. Archives of Disease in Childhood. 2011;96:619-21.
5. Johal HK, Danbury C. Conflict before the courtroom: challenging cognitive biases in critical decision-making. Journal of Medical Ethics. 2020:medethics-2020-106177.
6. Azoulay E, Timsit JF, Sprung CL. Prevalence and factors of intensive care unit conflicts: the conflicus study. Am J Respir Crit Care Med. 2009;180:853-60.
7. Flannery L, Ramjan LM, Peters K. End-of-life decisions in the intensive care unit (ICU) - exploring the experiences of ICU nurses and doctors - a critical literature review. Aust Crit Care. 2016;29(2):97-103.
8. Austin L, Huxtable R. Resolving disagreements about the care of critically ill children: Evaluating existing processes and setting the research agenda. Parental rights, best interests and significant harms: Medical decision-making on behalf of children post-Great Ormond Street Hospital v Yates: Hart Publishing; 2019.
9. Mehter HM, McCannon JB, Clark JA, Wiener RS. Physician approaches to conflict with families surrounding end-of-life decision-making in the intensive care unit. A qualitative study. Ann Am Thorac Soc. 2018;15(2):241-9.
10. Braunack-Mayer A. What makes a problem an ethical problem? An empirical perspective on the nature of ethical problems in general practice. Journal of Medical Ethics. 2001;27:98-103.
11. Guyatt G, Cairns J, Churchill D, Cook D, Haynes B, Hirsh J, et al. Evidence-based medicine: A new approach to teaching the practice of medicine. JAMA. 1992;268(17):2420-5.
12. Huxtable R, Ives J. Mapping, framing, shaping: a framework for empirical bioethics research projects. BMC Medical Ethics. 2019;20(1):1-8.
13. Ives J. A method of reflexive balancing in a pragmatic, interdisciplinary and reflexive bioethics. Bioethics. 2014;28(6):302-12.

For peer review only - http://bmjopen.bmj.com/site/about/guidelines.xhtml
For peer review only

14. Olmstead JA, Dahnke MD. The need for an effective process to resolve conflicts over medical futility: A case study and analysis. Critical Care Nurse. 2016;36(6):13-23.
15. Shamseer L, Moher D, Clarke M, Ghersi D, Liberati A, Petticrew M, et al. Preferred reporting items for systematic review and meta-analysis protocols (PRISMA-P) 2015 statement. Syst Rev. 2015;4(1).
16. Strech D, Synofzik M, Marckmann G. Systematic reviews of empirical bioethics. Journal of Medical Ethics. 2008;34:472-7.
17. Taylor H. What are 'best interests'? A critical evaluation of 'best interests' decision-making in clinical practice. Medical Law Review. 2016;24(2):176-205.
18. Schram A, Hougham G, Meltzer D, Ruhnke G. Palliative care in critical care settings: A systematic review of communication-based competencies essential for patient and family satisfaction. American Journal of Hospice & Palliative Medicine. 2017;34(9):887-95.
19. Salvador-Oliván JA, Marco-Cuenca G, Arquero-Avilés R. Errors in search strategies used in systematic reviews and their effects on information retrieval. Journal of the Medical Library Association. 2019;107(2).
20. Mahood Q, Eerd DV, Irvin E. Searching for grey literature for systematic reviews: challenges and benefits. Research Synthesis Methods. 2013;5(3):221-34.
21. Moher D, Liberati A, Tetzlaff J, Altman D. Preferred reporting items for systematic Reviews and meta-analyses: The PRISMA statement. Open Med. 2009;3(3):123-30.
22. Hong Q, Pluye P, Fàbregues S, Bartlett G, Boardman F, Cargo M, et al. Mixed Methods Appraisal Tool (MMAT), version 2018. Registration of Copyright (#1148552), Canadian Intellectual Property Office, Industry Canada.
23. Dixon-Woods M, Sutton A, Shaw R, Miller T, Smith J, Young B, et al. Appraising qualitative research for inclusion in systematic reviews: a quantitative and qualitative comparison of three methods. J Health Serv Res Policy. 2007;12(1):42-7.
24. Popay J, Roberts H, Sowden A, Petticrew M, Arai L, Rodgers M, et al. Guidance on the conduct of narrative synthesis in systematic reviews. Product from the ESRC Methods Programme. 2006;1:b92.
25. Schofield G, Brangan E, Dittborn M, Huxtable R, Selman L. Real-world ethics in palliative care: protocol for a systematic review of the ethical challenges reported by specialist palliative care practitioners in their clinical practice. BMJ Open. 2019;9:e028480:doi:10.1136/bmjopen-2018-028480.
26. Thomas J, Harden A. Methods for the thematic synthesis of qualitative research in systematic reviews. BMC Medical Research Methodology. 2008;8(45).
27. Lewin S, Booth A, Glenton C, Munthe-Kaas H, Rashidian A, Wainwright M, et al. Applying GRADE-CERQual to qualitative evidence synthesis findings: introduction to the series. Implementation Science. 2018;13(2):1-10.
28. Programme CAS. CASP Qualitative Checklist 2018 [Available from: https://casp-uk.net/wp-content/uploads/2018/01/CASP-Qualitative-Checklist-2018.pdf.

For peer review only

For peer review only - http://bmjopen.bmj.com/site/about/guidelines.xhtml

Ovid MEDLINE

1. exp Terminal Care/
2. Decision Making/
3. Decision Making, Shared/
4. exp Refusal to Treat/
5. Withholding Treatment/
6. Terminally Ill/
7. Palliative Care/
8. end of life.ti,ab,kf.
9. (end adj2 life).ti,ab,kf.
10. (end adj2 life care).ti,ab,kf.
11. EOL*.ti,ab,kf.
12. EOL care.ti,ab,kf.
13. best interest*.ti,ab,kf.
14. withdraw*.ti,ab,kf.
15. withhold*.ti,ab,kf.
16. decision*.ti,ab,kf.
17. resol*.ti,ab,kf.
18. consent.ti,ab,kf.
19. refusal*.ti,ab,kf.
20. Intensive Care Units/
21. critical care*.ti,ab,kf.
22. intensive care*.ti,ab,kf.
23. adult ICU.ti,ab,kf.
24. AICU.ti,ab,kf.
25. ICU*.ti,ab,kf.
26. "Dissent and Disputes"/
27. Negotiating/
28. Consensus/
29. conflict*.ti,ab,kf.
30. dispute*.ti,ab,kf.
31. disagreement*.ti,ab,kf.
32. dissent*.ti,ab,kf.
33. Physicians/
34. physician*.ti,ab,kf.
35. doctor*.ti,ab,kf.
36. nurse*.ti,ab,kf.
37. healthcare professional*.ti,ab,kf.
38. (health adj2 care professional).ti,ab,kf.
39. clinician*.ti,ab,kf.
40. famil*.ti,ab,kf.
41. relative*.ti,ab,kf.
42. therap*.ti,ab,kf.
43. surrogate*.ti,ab,kf.
44. prox*.ti,ab,kf.
45. patient*.ti,ab,kf.
46. Qualitative Research/
47. qualitative.ti,ab,kf.
48. interview*.ti,ab,kf.
49. focus group*.ti,ab,kf.
50. survey*.ti,ab,kf.
51. questionnaire*.ti,ab,kf.
52. empirical*.ti,ab,kf.
53. framework*.ti,ab,kf.
54. 1 or 2 or 3 or 4 or 5 or 6 or 7 or 8 or 9 or 10 or 11 or 12 or 13 or 14 or 15 or 16 or 17 or 18 or 19
55. 20 or 21 or 22 or 23 or 24 or 25
56. 26 or 27 or 28 or 29 or 30 or 31 or 32
57. 33 or 34 or 35 or 36 or 37 or 38 or 39 or 40 or 41 or 42 or 43 or 44 or 45
58. 46 or 47 or 48 or 49 or 50 or 51 or 52 or 53
59. 54 and 55 and 56 and 57 and 58

For peer review only

Ovid EMBASE

1. exp Terminal Care/
2. Decision Making/
3. Decision Making, Shared/
4. exp Patient Abandonment/
5. Treatment Withdrawal/
6. Terminally Ill Patient/
7. Palliative Therapy/
8. end of life.ti,ab,kf.
9. (end adj2 life).ti,ab,kf.
10. (end adj2 life care).ti,ab,kf.
11. EOL*.ti,ab,kf.
12. EOL care.ti,ab,kf.
13. best interest*.ti,ab,kf.
14. withdraw*.ti,ab,kf.
15. withhold*.ti,ab,kf.
16. decision*.ti,ab,kf.
17. resol*.ti,ab,kf.
18. consent.ti,ab,kf.
19. refusal*.ti,ab,kf.
20. Intensive Care Unit/
21. critical care*.ti,ab,kf.
22. intensive care*.ti,ab,kf.
23. adult ICU.ti,ab,kf.
24. AICU.ti,ab,kf.
25. ICU*.ti,ab,kf.
26. Conflict/
27. Negotiation/
28. Consensus/
29. conflict*.ti,ab,kf.
30. dispute*.ti,ab,kf.
31. disagreement*.ti,ab,kf.
32. dissent*.ti,ab,kf.
33. Physician/
34. physician*.ti,ab,kf.
35. doctor*.ti,ab,kf.
36. nurse*.ti,ab,kf.
37. healthcare professional*.ti,ab,kf.
38. (health adj2 care professional).ti,ab,kf.
39. clinician*.ti,ab,kf.
40. famil*.ti,ab,kf.
41. relative*.ti,ab,kf.
42. therap*.ti,ab,kf.
43. surrogate*.ti,ab,kf.
44. prox*.ti,ab,kf.
45. patient*.ti,ab,kf.
46. Qualitative Research/
47. qualitative.ti,ab,kf.
48. interview*.ti,ab,kf.
49. focus group*.ti,ab,kf.
50. survey*.ti,ab,kf.
51. questionnaire*.ti,ab,kf.
52. empirical*.ti,ab,kf.
53. framework*.ti,ab,kf.
54. 1 or 2 or 3 or 4 or 5 or 6 or 7 or 8 or 9 or 10 or 11 or 12 or 13 or 14 or 15 or 16 or 17 or 18 or 19
55. 20 or 21 or 22 or 23 or 24 or 25
56. 26 or 27 or 28 or 29 or 30 or 31 or 32
57. 33 or 34 or 35 or 36 or 37 or 38 or 39 or 40 or 41 or 42 or 43 or 44 or 45
58. 46 or 47 or 48 or 49 or 50 or 51 or 52 or 53
59. 54 and 55 and 56 and 57 and 58

For peer review only

Ovid PsycINFO

1. Decision Making/
2. exp Treatment Refusal/
3. Treatment Withholding/
4. Treatment Termination/
5. Terminally Ill Patients/
6. Palliative Care/
7. end of life.tw.
8. (end adj2 life).tw.
9. (end adj2 life care).tw.
10. EOL*.tw.
11. EOL care.tw.
12. best interest*.tw.
13. withdraw*.tw.
14. withhold*.tw.
15. decision*.tw.
16. resol*.tw.
17. consent.tw.
18. refusal*.tw.
19. Intensive Care/
20. critical care*.tw.
21. intensive care*.tw.
22. adult ICU.tw.
23. AICU.tw.
24. ICU*.tw.
25. Conflict/
26. Conflict Resolution/
27. Negotiation/
28. conflict*.tw.
29. dispute*.tw.
30. disagreement*.tw.
31. dissent*.tw.
32. Physicians/
33. physician*.tw.
34. doctor*.tw.
35. nurse*.tw.
36. healthcare professional*.tw.
37. (health adj2 care professional).tw.
38. clinician*.tw.
39. famil*.tw.
40. relative*.tw.
41. therap*.tw.
42. surrogate*.tw.
43. prox*.tw.
44. patient*.tw.
45. Qualitative Methods/
46. qualitative.tw.
47. interview*.tw.
48. focus group*.tw.
49. survey*.tw.
50. questionnaire*.tw.
51. empirical*.tw.
52. framework*.tw.
53. 1 or 2 or 3 or 4 or 5 or 6 or 7 or 8 or 9 or 10 or 11 or 12 or 13 or 14 or 15 or 16 or 17 or 18
54. 19 or 20 or 21 or 22 or 23 or 24
55. 25 or 26 or 27 or 28 or 29 or 30 or 31
56. 32 or 33 or 34 or 35 or 36 or 37 or 38 or 39 or 40 or 41 or 42 or 43 or 44
57. 45 or 46 or 47 or 48 or 49 or 50 or 51 or 52
58. 53 and 54 and 55 and 56 and 57

For peer review only

Web of Science

((((TS=(terminal care OR decision$making OR end NEAR/2 life OR end NEAR/2 life care OR palliative care OR terminal* ill* OR best interest* OR withdraw* OR withhold* OR decision* OR resol* OR consent OR refusal* OR EOL* OR EOL care))

AND TS=(dissent* OR dispute* OR conflict* OR disagreement* OR negotiat* OR consensus))

AND TS=(physician* OR doctor* OR nurse* OR health$care professional* OR clinician* OR famil* OR relative* OR therap* OR surrogate* OR prox* OR patient*))

AND TS=(intensive care* OR critical care* OR adult ICU* OR ICU* OR AICU))

AND TS=(qualitative* OR interview* OR focus group* OR survey* OR questionnaire* OR empirical* OR framework*)

Scopus

(TITLE-ABS-KEY(terminal care) OR TITLE-ABS-KEY(decision making) OR TITLE-ABS-KEY(end W/2 life) OR TITLE-ABS-KEY(end W/2 life care) OR TITLE-ABS-KEY(palliative care) OR TITLE-ABS-KEY(terminal* ill*) OR TITLE-ABS-KEY(best interest*) OR TITLE-ABS-KEY(withdraw*) OR TITLE-ABS-KEY(withhold*) OR TITLE-ABS-KEY(decision*) OR TITLE-ABS-KEY(resol*) OR TITLE-ABS-KEY(consent) OR TITLE-ABS-KEY(refusal*) OR TITLE-ABS-KEY(EOL*) OR TITLE-ABS-KEY(EOL care))

AND (TITLE-ABS-KEY(intensive care*) OR TITLE-ABS-KEY(critical care*) OR TITLE-ABS-KEY(adult ICU*) OR TITLE-ABS-KEY(ICU*) OR TITLE-ABS-KEY(AICU))

AND (TITLE-ABS-KEY(dissent*) OR TITLE-ABS-KEY(dispute*) OR TITLE-ABS-KEY(conflict*) OR TITLE-ABS-KEY(disagreement*) OR TITLE-ABS-KEY(consensus*) OR TITLE-ABS-KEY(negotiat*))

AND (TITLE-ABS-KEY(physician*) OR TITLE-ABS-KEY(doctor*) OR TITLE-ABS-KEY(nurse*) OR TITLE-ABS-KEY(healthcare professional*) OR TITLE-ABS-KEY(health care professional*) OR TITLE-ABS-KEY(clinician*) OR TITLE-ABS-KEY(famil*) OR TITLE-ABS-KEY(relative*) OR TITLE-ABS-KEY(therap*) OR TITLE-ABS-KEY(surrogate*) OR TITLE-ABS-KEY(prox*) OR TITLE-ABS-KEY(patient*))

AND (TITLE-ABS-KEY(qualitative*) OR TITLE-ABS-KEY(interview*) OR TITLE-ABS-KEY(focus group*) OR TITLE-ABS-KEY(survey*) OR TITLE-ABS-KEY(questionnaire*) OR TITLE-ABS-KEY(empirical*) OR TITLE-ABS-KEY(framework*))

CINAHLS

(MH “Terminal Care+” OR MH “Decision Making+” OR MH “Refusal to Treat+” OR MH “Treatment Refusal+” OR MH “Euthanasia, Passive” OR MH “Terminally Il Patients” OR MH “Palliative Care” OR TI (“end of life” OR “end W2 life” OR “end W2 life care” OR “EOL*” OR “EOL care” OR “best interest*” OR “withdraw*” OR “withhold*” OR “decision*” OR “resol*” OR “consent” OR “refusal*”) OR AB (“end of life” OR “end W2 life” OR “end W2 life care” OR “EOL*” OR “EOL care” OR “best interest*” OR “withdraw*” OR “withhold*” OR “decision*” OR “resol*” OR “consent” OR “refusal*”))

AND (MH “Critical Care” OR TI (“critical care*” OR “intensive care*” OR “adult ICU” OR “AICU” OR “ICU*”) OR AB (“critical care*” OR “intensive care*” OR “adult ICU” OR “AICU” OR “ICU*”))

AND (MH "Conflict Management“ OR MH “Negotiation” OR MH “Consensus” OR TI (“conflict*” OR “dispute*” OR “disagreement*” OR “dissent*”) OR AB (“conflict*” OR “dispute*” OR “disagreement*” OR “dissent*”))

AND (MH “Physicians+” OR TI (“physician*” OR “doctor*” OR “nurse*” OR “healthcare professional*” OR “health W2 care professional” OR “clinician*” OR “famil*” OR “relative*” OR “therap*” OR “surrogate*” OR “prox*” OR “patient*”) OR AB (“physician*” OR “doctor*” OR “nurse*” OR “healthcare professional*” OR “health W2 care professional” OR “clinician*” OR “famil*” OR “relative*” OR “therap*” OR “surrogate*” OR “prox*” OR “patient*”))

AND (MH “Qualitative Studies” OR TI (“qualitative” OR “interview*” OR “focus group*” OR “survey*” OR “questionnaire*” OR “empirical*” OR “framework*”) OR AB (“qualitative” OR “interview*” OR “focus group*” OR “survey*” OR “questionnaire*” OR “empirical*” OR “framework*”))

For peer review only

Project MUSE

(terminal* OR decision* OR making OR end OR life OR care OR palliative OR ill* OR best interest* OR withdraw* OR withhold* OR resol* OR consent OR refusal* OR EOL*)

AND (dissent* OR dispute* OR conflict* OR disagreement* OR negotiat* OR consensus)

AND (physician* OR doctor* OR nurse* OR health* OR professional* OR clinician* OR famil* OR relative* OR therap* OR surrogate* OR prox* OR patient*)

AND (intensive OR critical OR care OR unit* OR adult* OR ICU* OR AICU)

AND (qualitative* OR interview* OR “focus group*” OR survey* OR questionnaire* OR empirical* OR framework*)

LILACS

(“Terminal Care/” OR “Decision Making/” OR “Refusal to Treat/” OR “Withholding Treatment/” OR “Terminally Ill/” OR “Palliative Care/” OR terminal$ OR decision$ OR making OR end OR life OR care OR palliative OR ill$ OR best interest$ OR withdraw$ OR withhold$ OR resol$ OR consent OR refusal$ OR EOL$) AND (“Conflict Resolution/” OR “Conflict/” OR dissent$ OR dispute$ OR conflict$ OR disagreement$ OR negotiat$ OR consensus) AND (“Physician/” OR physician$ OR doctor$ OR nurse$ OR health$ OR professional$ OR clinician$ OR famil$ OR relative$ OR therap$ OR surrogate$ OR prox$ OR patient$) AND (“Intensive Care/” OR “Intensive Care Unit/” OR intensive OR critical OR care OR unit$ OR adult$ OR ICU$ OR AICU) AND (“Qualitative/” OR qualitative$ OR interview$ OR focus OR group$ OR survey$ OR questionnaire$ OR empirical$ OR framework$) [Subject descriptor]

OR (“Terminal Care/” OR “Decision Making/” OR “Refusal to Treat/” OR “Withholding Treatment/” OR “Terminally Ill/” OR “Palliative Care/” OR terminal$ OR decision$ OR making OR end OR life OR care OR palliative OR ill$ OR best interest$ OR withdraw$ OR withhold$ OR resol$ OR consent OR refusal$ OR EOL$) AND (“Conflict Resolution/” OR “Conflict/” OR dissent$ OR dispute$ OR conflict$ OR disagreement$ OR negotiat$ OR consensus) AND (“Physician/” OR physician$ OR doctor$ OR nurse$ OR health$ OR professional$ OR clinician$ OR famil$ OR relative$ OR therap$ OR surrogate$ OR prox$ OR patient$) AND (“Intensive Care/” OR “Intensive Care Unit/” OR intensive OR critical OR care OR unit$ OR adult$ OR ICU$ OR AICU) AND (“Qualitative/” OR qualitative$ OR interview$ OR focus OR group$ OR survey$ OR questionnaire$ OR empirical$ OR framework$) [Title words]

For peer review only

Reporting checklist for protocol of a systematic review and meta analysis.

Based on the PRISMA-P guidelines.

		Reporting Item	Page Number
**Title**			
Identification	#1a	Identify the report as a protocol of a systematic review	5
Update	#1b	If the protocol is for an update of a previous systematic review, identify as such	n/a
**Registration**			
	#2	If registered, provide the name of the registry (such as PROSPERO) and registration number	1
**Authors**			
Contact	#3a	Provide name, institutional affiliation, e-mail address of all protocol authors; provide physical mailing address of corresponding author	1
Contribution	#3b	Describe contributions of protocol authors and identify the guarantor of the review	14
**Amendments**			
	#4	If the protocol represents an amendment of a previously completed or published protocol, identify as such and list changes; otherwise, state plan for documenting important protocol amendments	13
**Support**			
Sources	#5a	Indicate sources of financial or other support for the review	14
Sponsor	#5b	Provide name for the review funder and / or sponsor	14
Role of sponsor or funder	#5c	Describe roles of funder(s), sponsor(s), and / or institution(s), if any, in developing the protocol	14

For peer review only

**Introduction**			
Rationale	#6	Describe the rationale for the review in the context of what is already known	4
Objectives	#7	Provide an explicit statement of the question(s) the review will address with reference to participants, interventions, comparators, and outcomes (PICO)	5
**Methods**			
Eligibility criteria	#8	Specify the study characteristics (such as PICO, study design, setting, time frame) and report characteristics (such as years considered, language, publication status) to be used as criteria for eligibility for the review	5
Information sources	#9	Describe all intended information sources (such as electronic databases, contact with study authors, trial registers or other grey literature sources) with planned dates of coverage	9
Search strategy	#10	Present draft of search strategy to be used for at least one electronic database, including planned limits, such that it could be repeated	8
Study records - data management	#11a	Describe the mechanism(s) that will be used to manage records and data throughout the review	10
Study records - selection process	#11b	State the process that will be used for selecting studies (such as two independent reviewers) through each phase of the review (that is, screening, eligibility and inclusion in meta-analysis)	10
Study records - data collection process	#11c	Describe planned method of extracting data from reports (such as piloting forms, done independently, in duplicate), any processes for obtaining and confirming data from investigators	10
Data items	#12	List and define all variables for which data will be sought (such as PICO items, funding sources), any pre-planned data assumptions and simplifications	10
Outcomes and prioritization	#13	List and define all outcomes for which data will be sought, including prioritization of main and additional outcomes, with rationale	n/a

For peer review only

Risk of bias in individual studies	#14	Describe anticipated methods for assessing risk of bias of individual studies, including whether this will be done at the outcome or study level, or both; state how this information will be used in data synthesis	11
Data synthesis	#15a	Describe criteria under which study data will be quantitatively synthesised	n/a
Data synthesis	#15b	If data are appropriate for quantitative synthesis, describe planned summary measures, methods of handling data and methods of combining data from studies, including any planned exploration of consistency (such as I2, Kendall's τ)	n/a
Data synthesis	#15c	Describe any proposed additional analyses (such as sensitivity or subgroup analyses, meta-regression)	n/a
Data synthesis	#15d	If quantitative synthesis is not appropriate, describe the type of summary planned	11
Meta-bias(es)	#16	Specify any planned assessment of meta-bias(es) (such as publication bias across studies, selective reporting within studies)	11
Confidence in cumulative evidence	#17	Describe how the strength of the body of evidence will be assessed (such as GRADE)	11

The PRISMA-P elaboration and explanation paper is distributed under the terms of the Creative Commons Attribution License CC-BY. This checklist was completed on 13. September 2021 using https://www.goodreports.org/, a tool made by the EQUATOR Network in collaboration with Penelope.ai

For peer review only
